# Efficacy of EHEC gold nanoparticle vaccines evaluated with the Shiga toxin-producing *Citrobacter rodentium* mouse model

**DOI:** 10.1128/spectrum.02261-23

**Published:** 2023-12-04

**Authors:** Sarah Bowser, Angela Melton-Celsa, Itziar Chapartegui-González, Alfredo G. Torres

**Affiliations:** 1 Department of Microbiology and Immunology, The University of Texas Medical Branch at Galveston, Galveston, Texas, USA; 2 Department of Microbiology and Immunology, Uniformed Services University of the Health Sciences, Bethesda, Maryland, USA; 3 Department of Pathology, The University of Texas Medical Branch, Galveston, Texas, USA; Cinvestav-IPN, Mexico City, Mexico

**Keywords:** EHEC, *Citrobacter rodentium*, nanovaccines, Shiga toxin, hemolytic uremic syndrome

## Abstract

Enterohemorrhagic *Escherichia coli* (EHEC) is a group of pathogenic bacteria responsible for several foodborne-associated outbreaks of human diarrheal disease. Although EHEC is a major cause of morbidity as well as the condition known as hemolytic uremic syndrome, linked to the production of Shiga toxins (Stx), there are no licensed vaccines approved for human use. Fully assessing vaccine efficacy against EHEC infections is challenging because conventional adult mice are inherently resistant to EHEC infection and do not develop hallmark symptoms of the disease. Therefore, in this study, we utilized a strain of the murine pathogen *Citrobacter rodentium* that has been lysogenized to produce Stx2d, which consistently causes lethal infection and other measurable disease outcomes following oral challenge, to further evaluate the protective efficacy of our previously formulated EHEC gold nanoparticle (AuNP) vaccines. Using this model, along with a non-Stx2d-producing *C. rodentium* strain, we assessed the protection conferred by AuNPs conjugated to three EHEC antigens: EscC, LomW, and intimin (Eae). We demonstrated that intranasally immunizing mice with AuNP-Eae provides partial protection against mortality caused by Stx2d-producing *C. rodentium* and that AuNP-EscC and AuNP-Eae moderately reduce intestinal burden of the non-Stx2d-producing *C. rodentium* strain compared with adjuvant-only-treated mice. Additionally, the AuNP-protein vaccines induced antigen- and pathogen-specific serum IgG and fecal IgA against both EHEC and *C. rodentium* while *in vitro* functional assays indicate that the elicited antibodies are bactericidal and prevent adherence of *C. rodentium* to intestinal epithelial cells. We propose that *C. rodentium* murine data could translate into correlates of protection.

**IMPORTANCE:**

Enterohemorrhagic *Escherichia coli* (EHEC) remains an important cause of diarrheal disease and complications worldwide, especially in children, yet there are no available vaccines for human use. Inadequate pre-clinical evaluation due to inconsistent animal models remains a major barrier to novel vaccine development. We demonstrate the usefulness of Stx2d-producing *Citrobacter rodentium* in assessing vaccine effectiveness because it more closely recapitulates human disease caused by EHEC.

## INTRODUCTION

While *Escherichia coli* is a Gram-negative bacterium that can typically be found as part of the normal intestinal microbiome in humans and animals, certain strains, known collectively as diarrheagenic *E. coli*, harbor virulence factors that are associated with disease (1). Globally, diarrheal disease is the second leading cause of mortality in children under the age of 5 and accounts for more than 1 billion cases and 500,000 deaths in this age group each year, primarily in developing countries (2). The Gates Foundation Global Enteric Multicenter Study (GEMS) found that one of the most common causes of diarrhea in these regions is pathogenic *E. coli* (3). The pathogroup enterohemorrhagic *E. coli* (EHEC) is a major cause of morbidity and is responsible for several major outbreaks worldwide, most notably associated with the serotype O157:H7, which can produce Shiga toxins (Stx) (4).

Humans acquire EHEC mainly through the consumption of contaminated food, water, and animal products (4). Once ingested, the bacterium utilizes two main virulence factors to colonize the gastrointestinal tract and cause disease (5). The first is a specialized Type 3 Secretion System (T3SS) encoded by a genomic pathogenicity island called the Locus of Enterocyte Effacement (LEE) that confers the ability to form attaching and effacing (A/E) lesions, a phenotype associated with colonization (5 – 7). Contact with the intestinal epithelial cells (IECs) allows the T3SS to inject certain effectors into the host cell, facilitating a close interaction between the pathogen and the IECs (5 – 7). This triggers changes in the host cell architecture, including rearrangement of the cytoskeleton and accumulation of actin beneath the attached bacterium (5 – 7). The second main virulence factor, which is also a defining feature of EHEC O157:H7 infections, is the production of Stx. These exotoxins are encoded within bacteriophages and their production is induced once the bacteria reach the intestines (5). Stx binds to Gb3 receptors on target cells and, once internalized, inhibits protein synthesis (8). These toxins, through a not fully understood mechanism, can reach circulation and travel to distant organs (9). If they reach the kidneys, damage to blood vessels can result in the potentially lethal condition called hemolytic uremic syndrome (HUS), characterized by a triad of microangiopathic hemolytic anemia, thrombocytopenia, and acute renal impairment (8, 10).

Following infection with EHEC, patients may develop self-resolving symptoms such as watery diarrhea that can progress to hemorrhagic colitis, abdominal pain, and low-grade fever (4). In 5%–10% of cases, the infection can progress to HUS, mostly in children under five and the elderly (4). In some regions, HUS can reach a mortality rate up to 10% (11). Treatment for EHEC infections is limited and mostly supportive because antibiotics are contraindicated due to their association with an increased risk of developing HUS (12). This makes the development of other treatment or preventative options imperative, such as vaccines, which are not currently available for human use.

There have been considerable efforts to develop a successful human vaccine to protect against EHEC; however, none have progressed to clinical trials (13, 14). Our lab previously developed vaccine formulations using spherical gold nanoparticles (AuNPs) conjugated to two immunogenic EHEC antigens—EscC, a T3SS structural protein encoded in the LEE, and LomW, a putative porin protein encoded by the Shiga toxin-producing 933 bacteriophage (15, 16). These antigens were discovered using an *in silico* bio- and immunoinformatics approach (17 – 19). Both subcutaneous (s.c.) and intranasal (i.n.) immunization with these formulations reduced intestinal colonization of BALB/c mice by EHEC following oral challenge and induced a robust, antigen-specific humoral response (15, 16). However, fully assessing their protective efficacy is challenging because EHEC does not reproduce human disease in mice. This highlights the importance of finding an animal model that more closely recapitulates EHEC-mediated disease and symptoms.

Although several animal models have been utilized to model EHEC infections, all have limitations and do not fully mimic disease as seen in humans (20, 21). Conventional, adult mice are inherently resistant to EHEC infection. For this reason, the pathogen *Citrobacter rodentium* has been employed as a model for infection. This pathogen is the causative agent of transmissible murine colonic hyperplasia and leads to disease manifestations such as weight loss, diarrhea, and even death in severe cases (22). Like EHEC, it also encodes for a conserved LEE T3SS and can form A/E lesions on IECs (23). Since the infection is usually self-resolving and has varying mortality among mouse strains, a strain of *C. rodentium* carrying the *stx2d* gene (ATCC DBS770) was constructed by Mallick et al., to enhance the EHEC-like features of this model (24). They showed that C57BL/6 mice orally challenged with this strain displayed significant weight loss and intestinal colonization and succumbed to infection by 10 days post-infection (dpi). They also exhibited histopathology of the large intestine and kidneys. However, mice challenged with an equivalent dose of the same *C. rodentium* strain but without the *stx2d* gene (ATCC DBS771) were also successfully colonized but only had modest weight loss without mortality or significant organ damage due to Stx2d. Therefore, in the present study, our goal was to further elucidate the protection conferred by our AuNP EHEC vaccines using these mouse models.

## RESULTS

### Infection of 10- to 12-week-old mice with Stx2d-producing *C. rodentium* results in lethal infection and measurable disease outcomes

**Fig 1:**
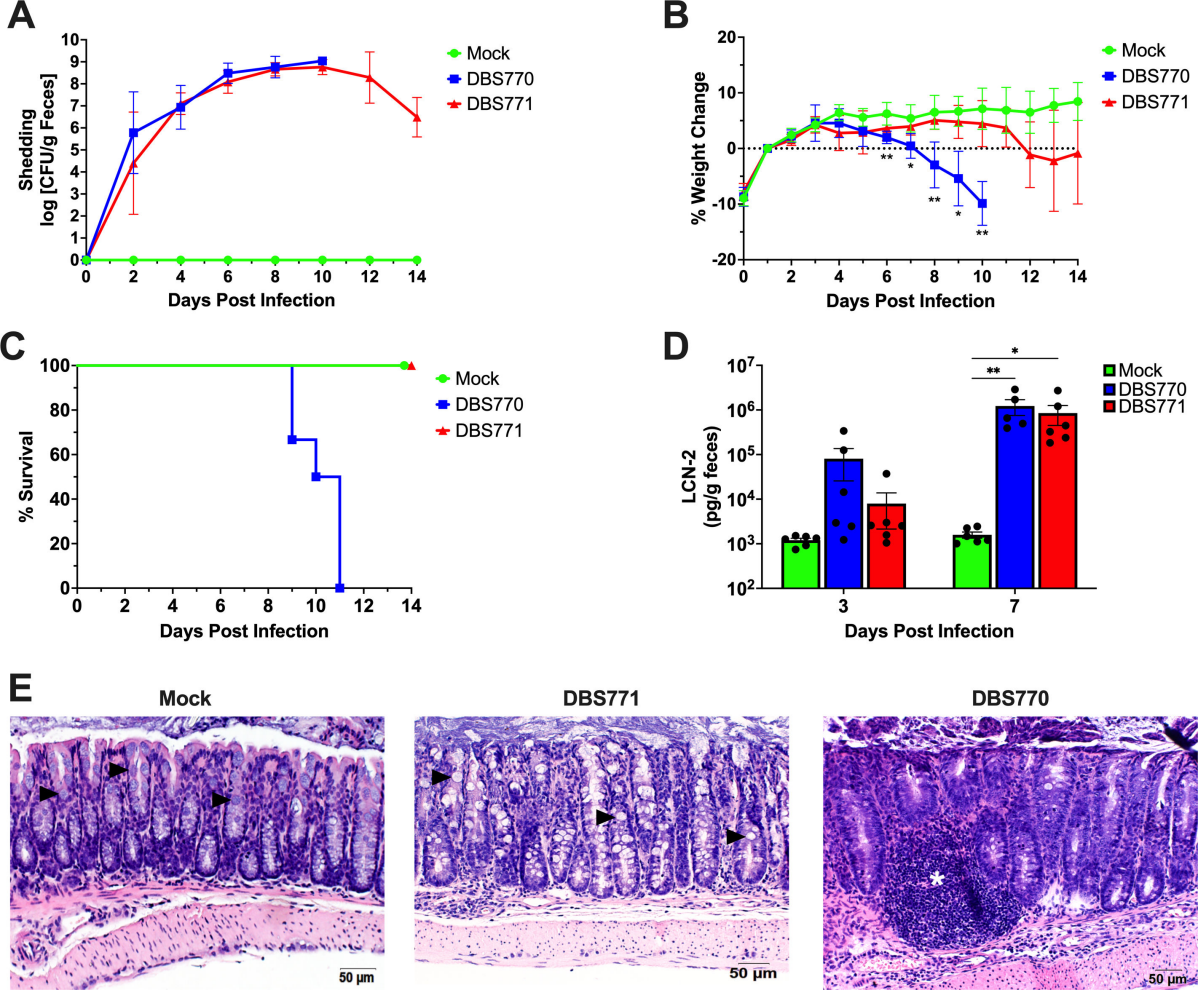
Disease outcomes after infection of 10- to 12-week-old mice with Stx2d-producing *C. rodentium* or Stx2d-negative *C. rodentium* via the feeding route. (**A**) Shedding of Stx2d-producing (DBS770) or Stx2d-negative (DBS771) *C. rodentium* in the feces of C57BL/6 mice. Counts were expressed as the log CFU/g of feces, and the average CFU (±SD) of six mice is shown. (**B**) Body weight changes before and following infection of mice, expressed as percentage change from day 1 post-infection (following recovery from 12-h fast prior to infection). Shown are the averages (±SD) of six mice. (**C**) Percentage survival of groups of *n* = 6 10–12-week-old mice that were infected with either DBS770 or DBS771 or mock infected. (**D**) LCN-2 concentrations in feces of mice that were infected with either DBS770 or DBS771 or mock infected. Each dot represents an individual mouse, and the average (±SEM) is shown. (**E**) Representative images of H&E-stained large intestines collected 11 days post-mortem (remaining DBS770-infected mouse) or 14 days following infection (remaining mock- or DBS771-infected mice). Black arrowheads indicate goblet cells that are abundant in mock- and DBS771-infected tissues but are depleted in intestines from DBS770-infected mice. The white asterisk indicates inflammatory infiltrate. Significant differences in weight changes were determined by mixed-effects analysis followed by Tukey’s multiple comparisons test, and significant differences in fecal LCN-2 concentrations were determined via two-way ANOVA followed by Tukey’s multiple comparisons test. *P ≤ 0.05 and **P ≤ 0.001.

Although Stx2d-producing *C. rodentium* was previously established to induce full lethality in young C57BL/6 mice, we sought to confirm the disease kinetics in the conditions of our animal facility, as well as verify that the model would elicit similar outcomes in older mice, considering the length of our vaccine regimens (24, 25). Therefore, we orally challenged 10- to 12-week-old female C57BL/6 mice with 1 × 10^9^ CFU of either Stx2d-producing *C. rodentium* strain (DBS770) or the Stx2d-negative (DBS771) strain (*n* = 6 for each group). Mice treated with phosphate-buffered saline (PBS) were used as a control (mock, *n* = 6). Furthermore, we utilized a feeding method of infection, which involves inoculating a small piece of rodent chow with the bacterial suspension and presenting it to fasted mice until consumption, because it was previously demonstrated that this method exhibits more consistent disease kinetics at even lower inoculum (25). Also, this technique is safer to administer, does not require anesthesia, and more closely resembles natural human infection with EHEC. Following infection, mice were monitored for up to 14 days, and feces were collected before infection and every 2 days after to both enumerate shedding of the bacteria and track colonization. Mice infected with either DBS770 or DBS771 increasingly shed the bacteria over 10 and 14 dpi, respectively (Fig. 1A); however, only DBS770 caused significant weight loss (Fig. 1B) and 100% lethality (Fig. 1C). Fecal concentrations of lipocalin-2 (LCN-2), which is an acute phase protein previously used to quantitatively measure intestinal inflammation following *C. rodentium* infection (23, 26, 27), were elevated at 3 dpi and significantly increased at 7 dpi in both DBS770- and DBS771-infected animals compared with the mock controls (Fig. 1D). Lastly, intestines removed from DBS771-infected mice at 14 dpi exhibited high bacterial burdens (Fig. S1). These organs showed little to no inflammatory damage or changes to the mucosa and overall normal intestinal architecture, while those from the remaining DBS770-infected mouse at 11 dpi displayed pockets of inflammatory infiltrates, sloughing and damage to the epithelia, lengthening of the crypts, and loss of mucous-producing goblet cells (Fig. 1E). Although both DBS770 and DBS771 were able to colonize C57BL/6 mice and cause inflammation, only mice infected with the Stx2d-producing strain had relevant morbidity and mortality. Nevertheless, disease manifestations resulting from either infection provide a dependable model to mimic EHEC disease, even in adult mice.

### Immunization of mice with AuNPs linked to EHEC antigens elicits antigen- and pathogen-specific humoral immune responses

**Fig 2:**
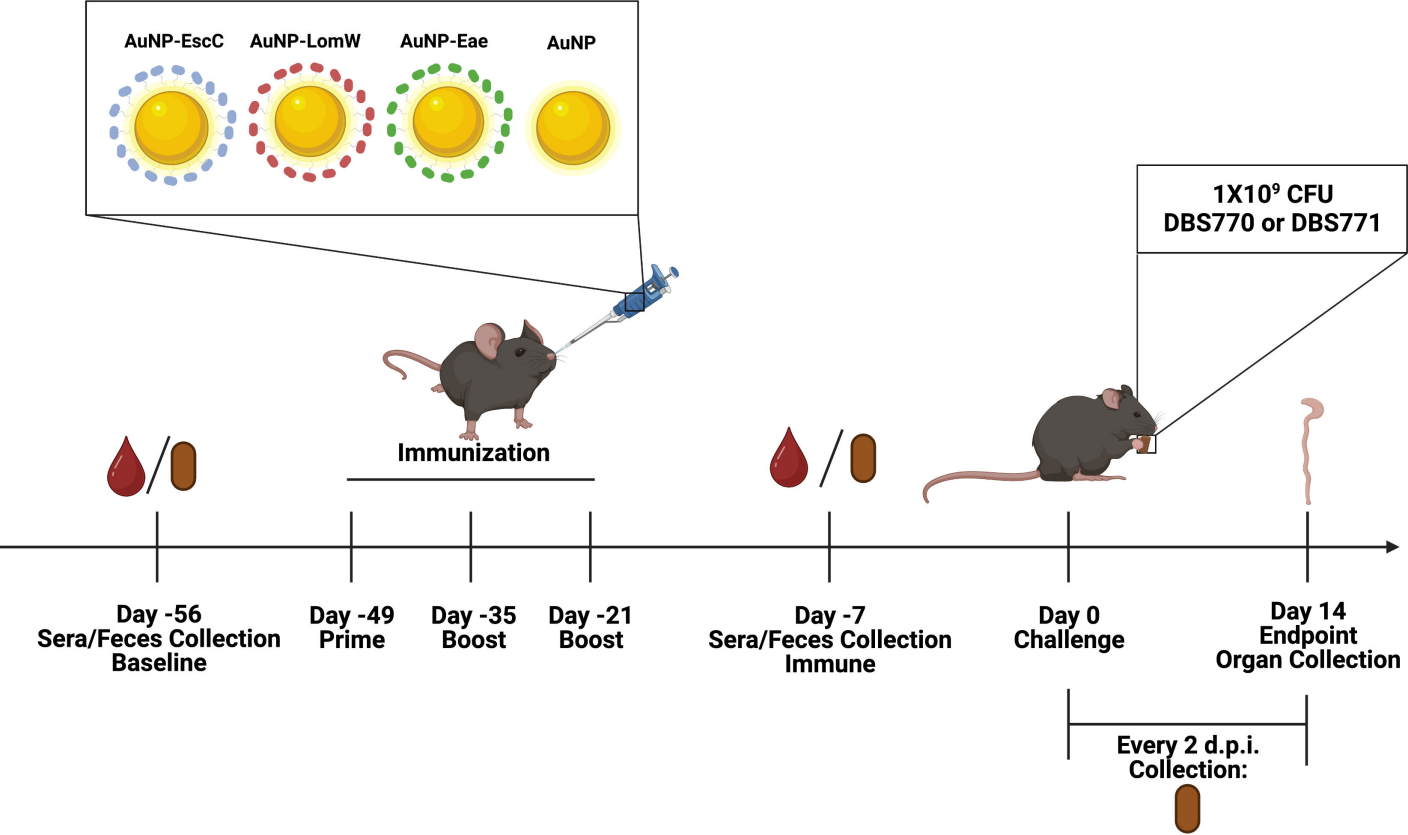
Timeline of vaccination schedule. The figure depicts the schedule of vaccination of C57BL/6 mice with AuNPs conjugated to the EHEC antigens and the subsequent infection with either DBS770 or DBS771 via the feeding route, along with blood, feces, and organ collection. A total of *n* = 24 mice were immunized with each vaccine formulation, and *n = 12* from each group were infected with DBS770 while the other *n* = 12 were infected with DBS771. Image was created using Biorender.com.

Although it was previously determined that our AuNP-protein vaccines elicit a protective immune response that reduces intestinal colonization of BALB/c mice by EHEC, infection with EHEC does not lead to any other symptoms of disease (15, 16). Therefore, we used the infection model with both Stx2d-producing and Stx2d-negative *C. rodentium* to further elucidate the protection offered by our vaccines. Using the same established immunization regimen used against EHEC in mice, 5- to 7-week-old female C57BL/6 mice were i.n. immunized three times at 2-week intervals with AuNPs chemically conjugated to one of three EHEC antigens: EscC, a T3SS structural protein encoded by the LEE; LomW, a putative outer membrane porin protein; and intimin (*eae*), a protein encoded in the LEE that is essential for A/E lesion formation and has been used in various other pre-clinical EHEC vaccine studies with some success (Fig. 2) (13, 28). The vaccine formulations contained a total of 5 µg of protein and 10 µg of the adjuvant cholera toxin B subunit, previously described to stimulate strong mucosal immune responses (29). Mice receiving unconjugated AuNPs plus the adjuvant were used as a control. Three weeks after the last immunization, mice were infected via feeding with 1 × 10^9^ CFU of either DBS770 or DBS771 and monitored for up to 14 days. Weight loss and mortality were monitored daily, while fecal samples were collected every 2 dpi to enumerate bacterial shedding. At 14 dpi, ceca and large intestines from the remaining mice were collected for either bacterial burden or histopathology.

**Fig 3:**
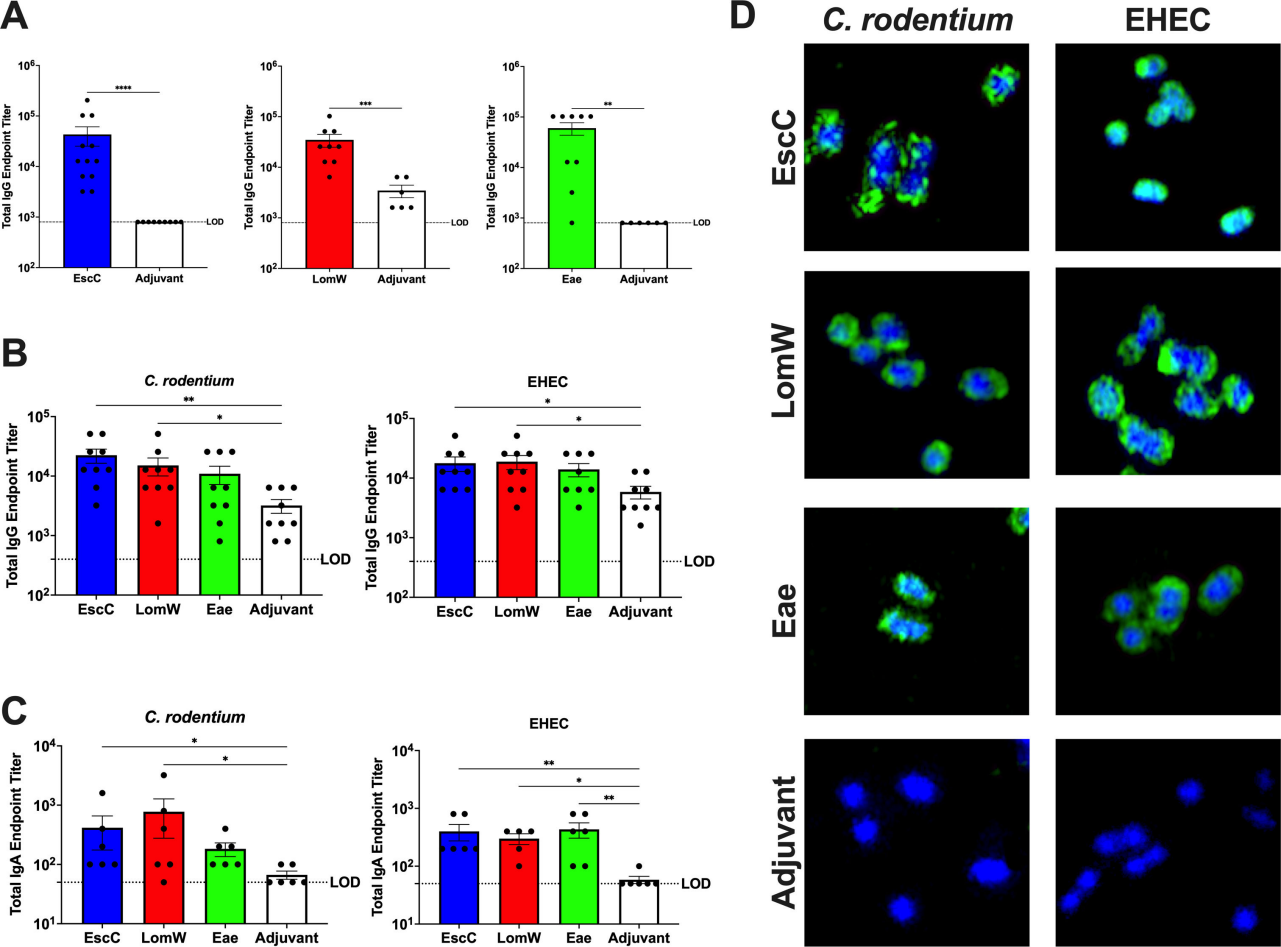
Humoral response following immunization with AuNP vaccines. (**A**) Antigen-specific serum IgG antibody titers were determined via ELISA with plates coated with individual antigens. (**B and C**) Pathogen-specific serum IgG (**B**) and fecal IgA (**C**) titers were determined by coating plates with lysates of individual pathogens. Endpoint titers are defined as the average +2SD of the levels measured for naïve sera. Each dot represents individual mice, and the averages (±SEM) are shown for all antibody data. (**D**) Micrographs showing cross-reactivity of antibodies in immune sera with both *C. rodentium* and EHEC; bacteria were incubated in the presence of heat-inactivated sera from AuNP-EscC-, AuNP-LomW-, or AuNP-Eae-immunized mice or from adjuvant-only-treated mice. Bacteria were stained with a goat anti-mouse IgG conjugated to Alexa-488 secondary antibody (green), and DNA was probed with DAPI (blue). Green indicates bound antibody to surface of bacterial cells. Significant differences in antigen-specific titers were determined by the Mann-Whitney non-parametric test, and significant differences in pathogen-specific titers were determined via the Kruskal-Wallis’s test followed by Dunn’s multiple comparisons test. *P ≤ 0.05, **P ≤ 0.01, ***P ≤ 0.001, and ****P ≤ 0.0001.

To evaluate both antigen- and pathogen-specific immune responses elicited by the AuNP vaccines, sera and feces collected prior to infection and 2 weeks following the last immunization were used to measure endpoint titers of IgG and IgA, respectively. The serum total IgG endpoint titers against the three antigens were significantly higher compared with adjuvant-only-treated animals (Fig. 3A), indicating the ability of all antigens to stimulate a robust, systemic humoral immune response in C57BL/6 mice. We also ensured that antibodies elicited by the EHEC antigens have cross-reactivity with *C. rodentium*. We found that AuNP-EscC- and AuNP-LomW-immunized mice exhibited significantly higher *C. rodentium*- and EHEC-specific total serum IgG (Fig. 3B) and fecal IgA (Fig. 3C) endpoint titers compared with the control group. A similar trend was seen in samples from AuNP-Eae-immunized mice (Fig. 3B and C), with a significant increase in EHEC-specific fecal IgA endpoint titers (Fig. 3C).

To corroborate the pathogen-specific ELISA results, immunofluorescence staining was used to visualize the antibodies binding to the surface of the bacteria. *C. rodentium* and EHEC were incubated in the presence of 10% heat-inactivated serum from AuNP-EscC-, AuNP-LomW-, or AuNP-Eae-immunized mice, as well as sera from adjuvant-only-treated animals. Antibodies in the sera from all AuNP-protein-immunized groups recognized the surface of both pathogens, with no evident recognition from the adjuvant-only group (Fig. 3D). Overall, the pathogen-specific immunological data indicate that the antibodies elicited by our vaccines have comparable reactivity against both pathogens, further justifying the use of Stx2d-producing *C. rodentium* as a model for EHEC.

### Antibodies elicited by the AuNP vaccines have bactericidal and neutralizing properties against *C. rodentium*

**Fig 4:**
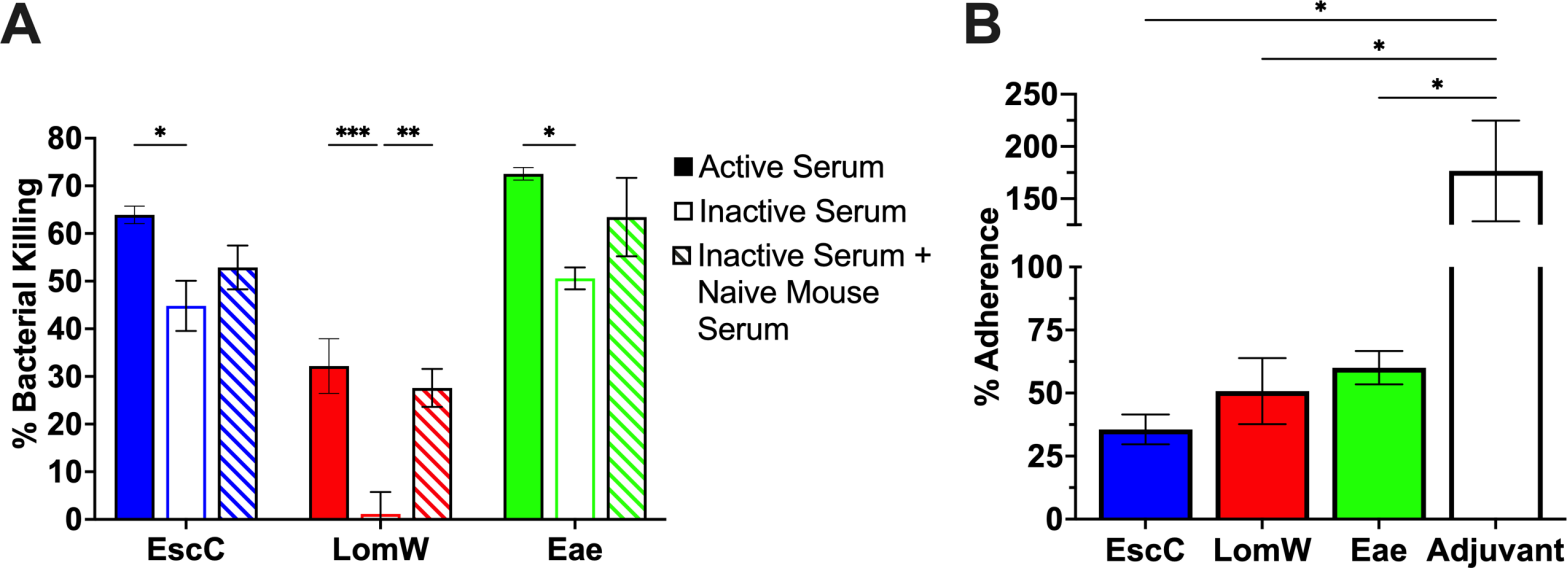
Functionality and cross-reactivity of serum antibodies elicited by the AuNP vaccines. (**A**) To assess bactericidal properties of immune sera, *C. rodentium* DBS770 (1 × 10^5^ CFU) was incubated with 10% active, inactive, or inactive plus exogenous complement sera. Bacterial killing was normalized using bacteria surviving after exposure to serum from adjuvant-only-treated mice. (**B**) For serum adherence inhibition, *C. rodentium* DBS770 (1 × 10^6^ CFU) was incubated in the presence of inactivated sera from AuNP-EscC-, AuNP-LomW-, or AuNP-Eae-immunized mice, or from adjuvant-only or naïve mice. After incubation, bacteria were used to infect monolayers of Caco-2 cells (MOI 10) for 2 h. Percentages of adhered bacteria were determined with the following equation: output CFU/input CFU × 100. Percentages were normalized to the adherence of bacteria in the presence of naïve sera. Serum bactericidal and adherence inhibition data are expressed as means (±SEM) of results from at least 2 independent experiments using pooled sera from *n* = 12 mice. Significant differences in bacterial killing were determined via two-way ANOVA followed by Tukey’s multiple comparisons test and via one-way ANOVA followed by Dunnett’s multiple comparison’s test for bacterial adherence. *P ≤ 0.05, **P ≤ 0.01, and ***P ≤ 0.001.

In past studies, our group demonstrated through *in vitro* methods that serum antibodies from mice immunized with the AuNP vaccines have both bactericidal and neutralizing properties against EHEC (15, 16). Therefore, to confirm that the antibodies would have similar functionality against *C. rodentium*, we evaluated its functionality against this murine pathogen. To first assess the bactericidal activity of serum antibodies by complement-mediated killing, *C. rodentium* DBS770 was incubated in the presence of active sera, heat-inactivated sera, or heat-inactivated sera plus naïve mouse sera to reconstitute complement. Bacteria were incubated with 10% sera from either AuNP-EscC-, AuNP-LomW-, or AuNP-Eae-immunized mice or from adjuvant-only-treated mice, for 1 h at 37°C, and the resulting viable bacterial CFU was determined. When bacteria were incubated with active serum from any of the three AuNP-immunized mouse groups, significantly increased bacterial killing was observed compared with bacteria incubated with heat-inactivated serum (Fig. 4A). This effect was mostly restored for the AuNP-LomW-inactivated serum when reconstituted with exogenous complement, with a similar tendency seen in the other two AuNP groups (Fig. 4A). These results indicate that the bactericidal activity of serum antibodies is antigen specific and that the classical complement pathway participates in this process.

Since binding of bacterial cells to IECs is essential for disease progression in both *C. rodentium* and EHEC infections, we evaluated whether serum antibodies could prevent adherence of *C. rodentium* to Caco-2 human epithelial cells (5, 23). Bacteria incubated in the presence of 10% heat-inactivated serum from AuNP-protein-immunized mice, adjuvant-only-treated mice, or naïve-serum mice, were used to infect monolayers of Caco-2 epithelial cells. The percentage of adhered bacteria was calculated and normalized to adherence in the presence of naïve sera. We found that sera from all three AuNP-immunized groups significantly reduced the adherence of *C. rodentium* to human epithelial cells compared with bacteria incubated with serum from the adjuvant-only group (Fig. 4B). This proves that antibodies in the serum can specifically bind to bacterial surface antigens and block this stage in the infectious process, which would be a crucial step in protection seen *in vivo*.

### Mice immunized with AuNP-Eae are partially protected from mortality and tissue damage following infection with Stx2d-producing *C. rodentium*

**Fig 5:**
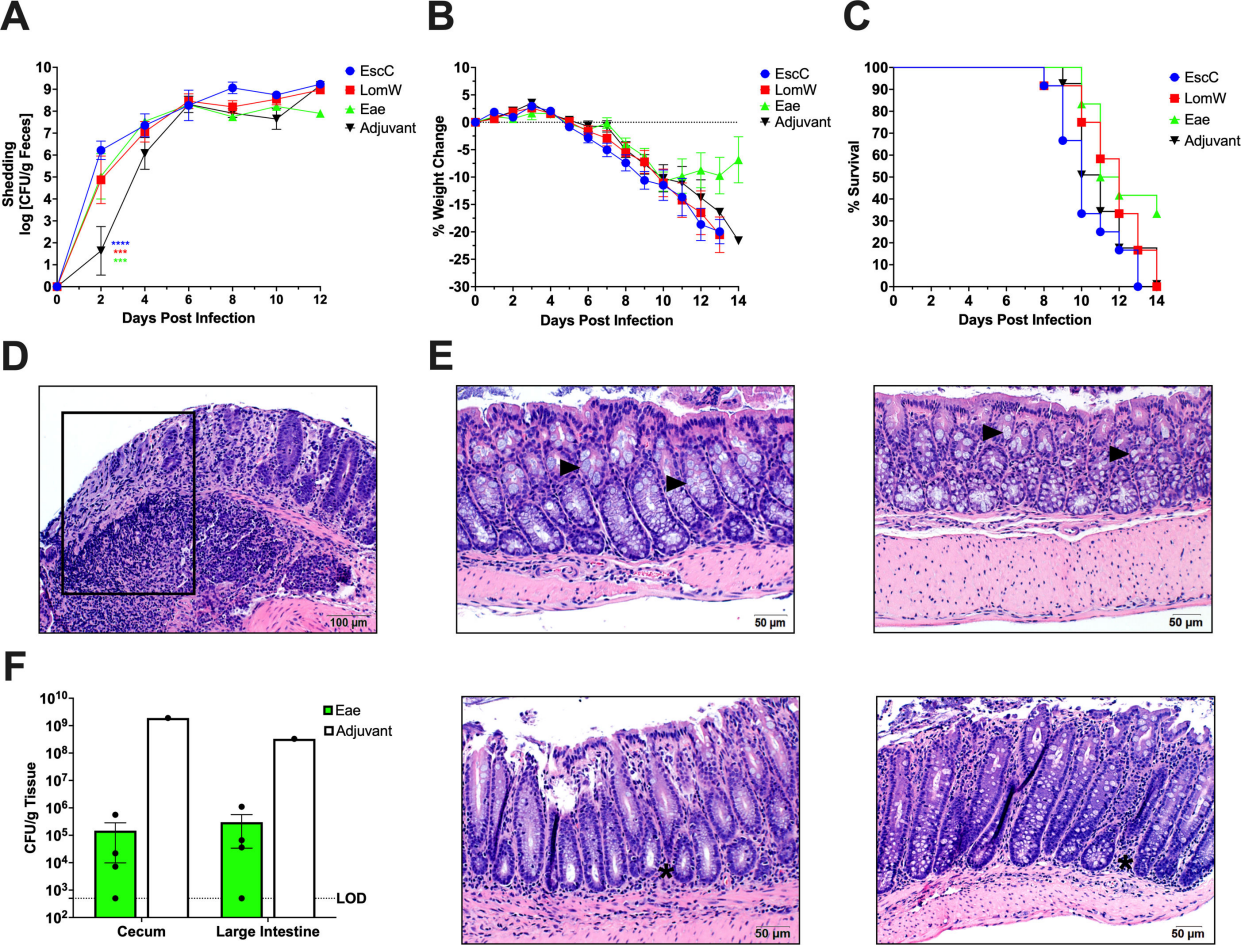
AuNP vaccine efficacy following infection with lethal DBS770. (**A**) Shedding of Stx2d-producing (DBS770) *C. rodentium* in the feces of AuNP-immunized mice was determined by collecting fecal samples before infection and every 2 days after infection. Counts were expressed as the log CFU/g of feces, and the average CFU (±SEM) of six mice is shown. Significant differences were determined by two-way ANOVA followed by Tukey’s multiple comparisons test. (**B**) Body weight changes before and following infection of immunized mice with DBS770, expressed as percentage change from before infection. Shown are the averages (±SEM) of 12 mice. Significant differences were determined by mixed-effects analysis followed by Tukey’s multiple comparisons test. (**C**) Percentage survival of groups of 12 AuNP immunized mice that were infected with DBS770. (**D**) H&E-stained large intestine collected on 14 dpi from the remaining adjuvant-only-treated mouse infected with DBS770. Black box indicates an area of crypt loss and inflammation. (**E**) H&E-stained large intestines from the four remaining mice that were immunized with AuNP-Eae and infected with DBS770, collected on 14 dpi. The top two panels are from mice whose organs show evidence of recovery from the infection, while the bottom two panels are from the mice that still displayed some inflammatory tissue damage. Black arrowheads show abundance of goblet cells and black asterisks indicate areas of inflammatory infiltrates. (**F**) Ceca and large intestines were collected from remaining AuNP-Eae and adjuvant-only treated mice on 14 dpi to evaluate intestinal burden. Viable counts were expressed as CFU/g of tissue, and the average CFU (±SEM) of 4 mice (AuNP-Eae-immunized) and representative 1 mouse (adjuvant-only treated) are shown. ***P ≤ 0.001 and ****P ≤ 0.0001.

Infection of C57BL/6 mice with DBS770 resulted in marked disease symptoms such as weight loss and severe intestinal tissue damage along with 100% lethality; therefore, we determined whether our AuNP vaccines could prevent these outcomes. Although there was initially a delay in fecal shedding of DBS770 in the adjuvant-only group following the feeding infection, mice in all AuNP-protein groups shed bacteria at a similar rate to the adjuvant-only-treated animals by 6 dpi (Fig. 5A). Mice in all groups began steadily declining in body weight at the same time, and all mice in the AuNP-EscC-, AuNP-LomW-, and adjuvant-only-treated groups succumbed to infection by day 14 (Fig. 5B and C). However, 4 of the 12 AuNP-Eae-immunized mice began recovering weight at 11 dpi and survived to the endpoint (Fig. 5B and C). Intestinal inflammation was also monitored by measuring fecal LCN-2 concentrations on 0, 2, 4, and 6 dpi, but there were no differences among the groups, with elevations beginning at 2 dpi (Fig. S2).

Next, we determined if the surviving mice had started to recover from other outcomes of the infection. Intestines taken from the four remaining AuNP-Eae-immunized mice were compared with the intestines taken from the last surviving adjuvant-only-treated animal, which had reached greater than 20% body weight loss on 14 dpi and was humanely euthanized. Histopathology of the large intestines from the adjuvant-only-treated mouse revealed extensive damage to the epithelia accompanied by inflammatory infiltrates, loss of goblet cells, and overall damage to the intestinal barrier (Fig. 5D). While the intestines of two of the surviving AuNP-Eae-immunized mice still had some infection-related pathology, such as epithelial damage, decrease in goblet cells, lengthening of the crypts, and inflammation (Fig. 5E, lower two panels), those from the other two surviving mice displayed normal colonic crypt lengths and numbers of goblet cells, along with intact epithelial layers and limited inflammatory cells (Fig. 5E, upper two panels). The histopathology results were substantiated with decreased cecal and large intestinal burdens seen in the four living AuNP-Eae-immunized mice compared with the remaining adjuvant-only treated animal (Fig. 5F). In fact, the two AuNP-Eae-immunized mice that exhibited the lowest organ burdens correlated with the reduced histopathology. Although the AuNP-EscC and AuNP-LomW vaccines did not protect against severe disease caused by Stx2d-producing DBS770, AuNP-Eae resulted in promising protection by preventing lethality in some mice and even allowed those mice to begin recovering from infection.

### Mice immunized with AuNP-proteins are moderately protected from intestinal burden following infection with Stx2d-negative *C. rodentium*

**Fig 6:**
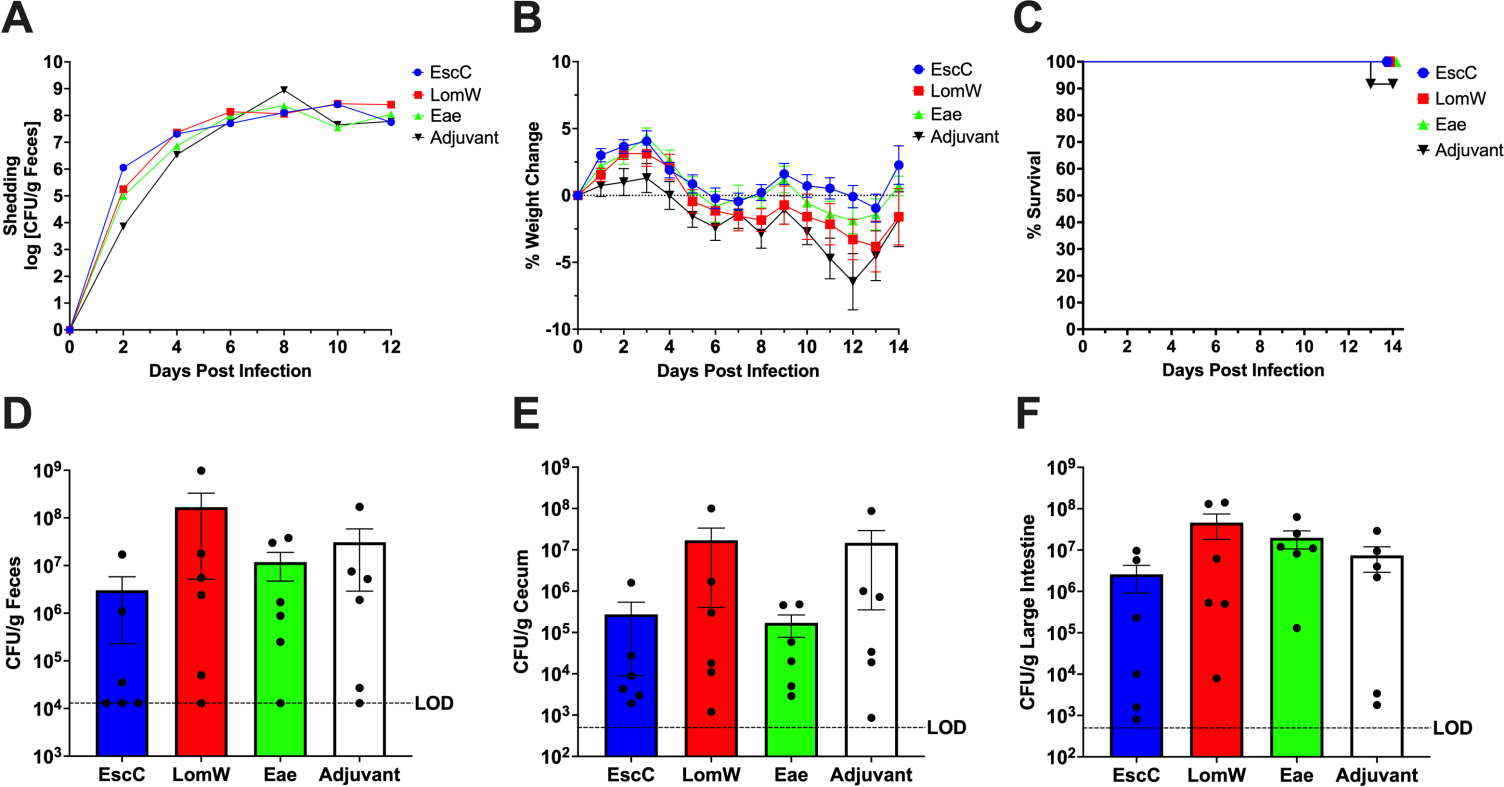
AuNP vaccine efficacy following infection with non-lethal DBS771. (**A**) Shedding of Stx2d-negative (DBS771) *C. rodentium* in the feces of AuNP-immunized mice was determined by collecting fecal samples. Counts were expressed as the log CFU/g of feces, and the average CFU (±SEM) of six mice is shown. Significant differences were determined by two-way ANOVA followed by Tukey’s multiple comparisons test. (**B**) Body weight changes before and following infection of AuNP-immunized mice with DBS771, expressed as percentage change from before infection. Shown are the averages (±SEM) of 12 mice. Significant differences were determined by two-way ANOVA followed by Tukey’s multiple comparisons test. (**C**) Percent survival of *n* = 12 AuNP-immunized mice that were infected with DBS771. (**D-F**) To evaluate fecal and organ burden with DBS771 at 14 dpi, feces (**D**), ceca (**E**), and large intestines (**F**) were collected from all remaining mice. Viable counts were expressed as CFU/g of feces or tissue, and the average CFU (±SEM) of six animals per group is shown. Significant differences in fecal and organ burdens were determined via one-way ANOVA followed by Dunnett’s multiple comparisons test.

Even though DBS771 does not lead to severe disease in C57BL/6 mice, its intestinal colonization abilities can still be used to measure vaccine efficacy. All AuNP-protein-immunized and adjuvant-only-treated mice shed bacteria and had similar weight fluctuations following feeding infection with DBS771 (Fig. 6A and B). One adjuvant-only-treated mouse did succumb to infection at 13 dpi while all other mice survived to the endpoint (Fig. 6C). Feces, ceca, and large intestines taken from AuNP-EscC-immunized animals at 14 dpi revealed some reduction in bacterial burden compared with the adjuvant-only-treated animals, but this was not significant (Fig. 6D through F). This trend was also found in AuNP-Eae-immunized mice, with modest decreases in fecal and cecal burdens (Fig. 6D through F). No reduction in bacterial burdens was seen in AuNP-LomW-immunized mice (Fig. 6D through F). Organs were collected from all groups to identify histopathological changes; however, there were no differences identified between AuNP-protein-vaccinated mice and adjuvant-only mice, mostly likely because this pathogen does not consistently cause severe tissue damage (data not shown). Lastly, we measured fecal LCN-2 concentrations on 0, 2, 4, and 6 dpi but there were no differences among the groups, with elevations beginning at 4 dpi (Fig. S2).

## DISCUSSION

According to the World Health Organization, pathogenic *E. coli* is one of the most important etiological agents of diarrheal disease in children, especially in low-income countries, and can even lead to malnutrition and stunted growth in these areas due to the lack of appropriate medical care (2). One of the most studied and notorious EHEC serotypes is EHEC O157:H7, which is responsible for many outbreaks all over the world (4). It is well established that this pathogen utilizes two main virulence factors, the production of potent Shiga toxins and the formation of A/E lesions on the epithelial layer of the large intestines (5). Following infection, EHEC can cause disease in humans that ranges from mild gastroenteritis to hemorrhagic colitis (5, 30). In susceptible populations (e.g., elderly or children), disease can progress to HUS, which can be lethal. Regardless of its burden on health care and well-characterized virulence characteristics, there are still no reliable treatment options for EHEC infections or prophylactic vaccines approved for human use. In fact, there have been no EHEC vaccines that have progressed into human clinical trials, despite considerable efforts using various platforms and a plethora of immunogenic antigens (13). A major reason for this is the lack of reliable animal models to perform pre-clinical screening (20, 21). While gnotobiotic piglets and infant rabbits have been used to model the characteristic A/E lesions by EHEC, there are important limitations in their breeding, handling, and use in long-term vaccination studies (31, 32). Mice offer the most obvious benefits in vaccine studies, but conventional wild-type mice do not consistently develop hallmark symptoms of disease following infection with EHEC. Wild-type BALB/c mice, for example, do develop some mortality and morbidity following a high-dose (10^9^ CFU) EHEC challenge, including renal damage (33). This is similar to disease seen in microbiome-deplete mouse models, such as streptomycin-treated or germ-free mice, which succumb to infection by EHEC (34 – 38). These models do not fully reflect human disease because infection is Stx mediated and does not require the development of A/E lesions. Therein lies the importance of using animal models that more closely recapitulate human disease in the progression of EHEC vaccine research.

The murine pathogen *C. rodentium* is often considered the mouse counterpart of EHEC because it leads to the development of the hallmark A/E lesions in mice and encodes a conserved LEE genomic pathogenicity island essential for virulence (22, 23). While this pathogen can be used as a model for EHEC infection in mice because it also leads to outward disease symptoms like diarrhea, weight loss, and even death, its utility is still limited due to its varying and inconsistent severity in different mouse strains (39 – 41). To improve upon this model, Mallick et al. engineered *C. rodentium* strain DBS100 to produce Stx2d (24). This strain was demonstrated to induce consistent, reproducible lethal disease in conventional mice at even lower infection inoculums (e.g., 3 × 10^4^ CFUs) than are typically used for enteric pathogens, and it provokes inflammatory tissue damage to the intestinal tract, as well as Stx-mediated renal damage. This model encompasses both major EHEC virulence factors while causing reliable, measurable disease outcomes in wild-type mice, which is the rationale behind our decision to apply it in our AuNP-based EHEC vaccine studies.

Previously, our lab developed AuNP-based vaccines for bacterial pathogens, including EHEC (15, 16, 42 – 44), which exhibited reduced colonization of BALB/c mice with EHEC and induced robust systemic and mucosal humoral immune responses This platform is desirable due to its general safety, ease in functionalization of molecules to their surface, and their ability to enhance uptake of antigens by antigen-presenting cells (45). Historically, EHEC vaccine studies focused on targeting adhesin antigens or Stx; however, the antigens selected for our EHEC vaccines—EscC and LomW—were discovered via *in silico* methods based on their location in the outer membrane, predicted T- and B-cell epitopes, and their absence in commensal *E. coli* strains (13, 17 – 19). Further characterization, and thus movement into clinical trials, is challenging because EHEC does not provide sufficient disease markers in the model to fully defend the efficacy of our AuNP vaccines.

Here, we demonstrated that the Stx2d-producing *C. rodentium*, which more closely recapitulates human intestinal disease as seen with EHEC, is a viable model to assess the effectiveness of novel vaccine candidates. Former studies with this pathogen, specifically using the feeding model of infection, utilized mice corresponding to 6 to 8 weeks of age (24, 25). To mitigate concerns regarding the age of mice used in our vaccine regimen, which equals around 8 full weeks starting from arrival at our facilities to infection, we initially evaluated this model in 10- to 12-week-old C57BL/6 female mice. Consistent disease kinetics were observed even at a more advanced age, allowing us to use the resulting symptoms to assess our AuNP vaccines (Fig. 1). Before completing the vaccine studies, we sought to ensure that the antibodies elicited by our selected antigens would have cross-reactivity and potential protection against *C. rodentium*. EscC and intimin produced by *C. rodentium* share a high amino acid sequence identity with those encoded by EHEC (96% and 78%, respectively); therefore, the significant pathogen-specific serum and fecal antibody titers elicited by these antigens were expected (Fig. 3B and C) (46). LomW, however, does not have a high identity to proteins encoded by *C. rodentium*. Through *blastp*, we found that the closest protein with sequence homology to LomW in *C. rodentium* is an Ail/Lom family protein, which only shares 32% identity to LomW. This could be the reason for the lack of protection against lethality or organ burden observed in animals vaccinated with this antigen. Surprisingly, however, we did observe reactivity of elicited antibodies with the surface of *C. rodentium* (Fig. 3D) but the specific antigens they are binding to warrant further investigation. This does present limitations to this model, as novel antigens selected for evaluation will also need to be encoded by *C. rodentium* to see full protective efficacy. Furthermore, this could be included as criteria for antigens selected via the same *in silico* methods previously utilized to identify EscC and LomW.

Protective immunity against EHEC at the interface of the intestinal epithelial cells involves neutralizing IgA antibodies, with IgG also playing a role (13, 47, 48). This immunity is important in blocking the initial adherence of the bacteria, essential for its pathogenesis. This differs with *C. rodentium*, however, because IgG has been reported to be more protective, with IgA not being required for clearance of *C. rodentium* (49). This could present challenges in using *C. rodentium* as a model when developing vaccines, considering eliciting strong IgA responses, while important for EHEC, is not an indication of protection in mice against *C. rodentium*. Therefore, it is apparent that vaccine studies using this model should elicit robust IgG and IgA. In our previous trials using AuNP-EscC and AuNP-LomW, we demonstrated that these antigens induce strong systemic and mucosal antibodies through different immunization routes in BABL/c mice (15, 16). We sought to recapitulate these findings in the present study using C57BL/6 mice. We found that both EscC and LomW, as well as intimin, induced antigen-specific serum IgG titers following intranasal vaccination. EscC and LomW also stimulated significant pathogen-specific serum IgG and fecal IgA (Fig. 3B and C). Although we only observed modest pathogen-specific antibody titers from intimin-immunized animals, through *in vitro* studies, we confirmed that serum antibodies from all AuNP-proteins groups bind to and have functionality against *C. rodentium*, including bactericidal capabilities and adherence inhibition to Caco-2 cells (Fig. 4). Our next step was to verify that these immune responses would be sufficient for protection.

We demonstrated in past vaccine studies that AuNPs conjugated to EscC or LomW, or in combination, significantly reduce EHEC colonization in BALB/c mice (15, 16). Our lab has not tested intimin in this context; however, other pre-clinical studies using both EHEC and *C. rodentium* as a model showed some protective efficacy with this LEE-encoded antigen (13, 28). Using both a lethal and non-lethal *C. rodentium* model, we confirmed that our AuNP-based vaccines do provide a trend toward protection against both (Fig. 5 and 6). The i.n. immunization with AuNP-EscC did modestly reduce both fecal and intestinal burden at 14 dpi following infection with DBS771, and AuNP-Eae protected 4 out of 12 mice from lethal infection with DBS770 (Fig. 5), with some reduction in fecal and cecal burden with DBS771 (Fig. 6). Even with moderate efficacy, we can still use the information collected from this vaccine study to establish future studies, which include optimizing our vaccine strategy to enhance protection, through other routes of immunization or using a combination of antigens. Nonetheless, we have more thoroughly evaluated the effectiveness of our EHEC vaccines using Stx2d-producing *C. rodentium* as a valuable tool. We hope this will open the door for more comprehensive pre-clinical testing, which will eventually pave the way for human trials.

## MATERIALS AND METHODS

### Bacterial strains and growth conditions

The *C. rodentium* strains used in this study were purchased from the American Type Culture Collection (ATCC). Stx2d-producing *C. rodentium* (ATCC DBS770) and non-Stx2-producing *C. rodentium* (ATCC DBS771) were both routinely grown aerobically at 37°C in Luria-Bertani (LB) broth supplemented with antibiotics—12.5 µg/mL chloramphenicol alone (DBS770) or 12.5 µg/mL chloramphenicol and 25 µg/mL kanamycin (DBS771)—unless otherwise stated. The *E. coli* strain used in this study, EHEC O157:H7 strain 86–24, was also routinely grown in LB broth. For mouse infections, DBS770 and DBS771 were processed as previously described, with minor changes (24, 25). One day prior to infection, DBS770 and DBS771 were each inoculated into 6 × 40 mL LB broth tubes with the appropriate antibiotics and incubated statically at 37°C in 5% CO_2_ until the cultures reached an optical density (OD)_600_ of ~0.6–0.7 (about 19.5 h). Each culture was pelleted at 4,000 × *g* for 30 min, resuspended in 500 µL sterile 1× PBS, pelleted again at 4,000 × *g* for 10 min, and then resuspended in 60 µL. All resulting cultures for each strain were combined into one inoculum, which was subsequently used for mouse infections. For *in vitro* studies, overnight cultures of *C. rodentium* strains and EHEC were diluted 1:20 in Dulbecco’s modified Eagle’s medium (DMEM) without fetal bovine serum (FBS) or antibiotics to express the type III secretion system components, as previously described (6). Cultures were incubated statically at 37°C for 4 h. For *in vivo* studies, after organ or feces homogenization, bacteria were plated onto MacConkey agar selective media.

### Cloning

EHEC (EDL933) DNA was isolated with a DNeasy Blood and Tissue Kit (Qiagen), following the manufacturer’s directions. To improve recombinant protein solubility, the N-terminus signal sequences of EscC (GenBank protein accession no. 12518466) and LomW (GenBank protein accession no. 12514345) were predicted using the SignalP 6.0 server, and the DNA segments without the predicted signal sequences were cloned into a pET30a(+) expression vector using NdeI and XhoI (New England BioLabs) restriction sites. The open reading frame for each protein was inserted *in frame* with a 6⨉His-tag on the C-terminus. Following ligation, pET30a(+)-EscC and pET30a(+)-LomW were transformed into DH5α competent *E. coli* cells following the manufacturer’s protocol (New England Biosciences). Upon confirmation of successful gene insertion via gel electrophoresis and directional sequencing (Azenta Life Sciences), plasmids were transformed into BL21 (DE3) competent *E. coli* cells (New England BioLabs) according to the manufacturer’s protocol.

### Protein purification and visualization

To induce EscC and LomW expression, overnight cultures were diluted 1:20 in 2 L of LB broth with 50 µg/mL kanamycin, grown to an OD_600_ of between 0.6 and 0.8, and induced with 1 mM (final concentration) of isopropyl β-D-1-thiogalactopyranoside (IPTG). Cultures were centrifuged at 4,000 × *g* for 20 min at 4 h post-induction, and the resulting bacterial pellets were kept at −80°C until use. The bacterial pellets were thawed and resuspended in 20 mL lysis buffer [PBS containing 10% glycerol, 25 mM sucrose, 1 mg/mL final concentration of lysozyme, and a tablet of cOmplete EDTA-protease inhibitor cocktail (Roche, Germany)]. The lysate was incubated on ice for 30 min, then sonicated, and centrifuged at 16,000 rpm for 45 min. The resulting pellet was used for subsequent washes with 0.5% Sarkosyl in lysis buffer to maximize soluble protein extraction. The soluble protein extracts were sterilized using a 0.2-µm pore size filter and then bound to nickel-NTA resin (Qiagen) affinity columns. The resin was washed with PBS buffer—10 mM imidazole, and proteins were eluted from the columns with a PBS buffer supplemented with 10% glycerol, 25 mM sucrose, and 250 mM imidazole. Fractions were collected and pooled, and imidazole was removed via dialysis overnight at 4°C in dialysis cassettes with 7,000 molecular weight cutoffs (MWCO) (Thermo Fisher Scientific). Endotoxin levels were evaluated using a Pierce LAL Chromogenic Endotoxin Quantification Kit (Thermo Fisher Scientific) following the manufacturer’s instructions. The purified proteins were quantified using bovine serum albumin (BSA) standards and a colorimetric bicinchoninic acid assay (BCA) using the manufacturer’s instructions and frozen at −20°C until use. For protein visualization, protein was run on SDS-PAGE gel by electrophoresis, and protein bands were visualized by staining with Coomassie blue stain, or gels were transferred to a nitrocellulose membrane for western blot analysis. The membranes were blocked overnight at 4°C in 5% skim milk in PBS with 0.05% Tween-20. A mouse anti-histidine antibody (1:5,000) was used to detect the C-terminus 6×His-tag, and horseradish peroxidase-conjugated rabbit anti-mouse IgG was used as a secondary antibody (Southern Biotech REF 6170–05). Protein bands were visualized by the addition of ECL substrate (Thermo Fisher Scientific), and the results were imaged with the Amersham Imager 600 (GE). Additionally, full-length intimin (Eae) gamma protein was provided to us by Dr. Angela Melton-Celsa. Its cloning and purification protocol was described previously (50).

### Coupling of proteins onto AuNPs

Spherical gold nanoparticles, 15 nm in diameter, were synthesized using the Turkevich method, as previously described (51). Briefly, 1 mM gold (III) chloride trihydrate was reduced with 90 mM sodium citrate dihydrate. Particle size and shape were confirmed using transmission electron microscopy. To stabilize conjugation of protein onto the AuNP surface, AuNPs were incubated with 0.1 mM polyethylene glycol (PEG)-3500 (Sigma-Aldrich) for 2 h. The particles were centrifuged at 16,000 × *g* and resuspended in 100 mM borate buffer with a 1-mM final concentration of DMTMM [4-(4,6-Dimethoxy-1,3,5-trizin-2-yl)−4-methylmorpholinium chloride] to activate the carboxylic acid end of the PEG-3500 linker. Following 1 h incubation, the AuNPs were pelleted and resuspended in 100 mM borate buffer containing the recombinant protein. Conjugation was conducted overnight at 4°C with constant rotation. The AuNPs were then pelleted and resuspended in 1× PBS. To confirm protein conjugation, AuNPs were run on SDS-PAGE gels with electrophoresis, and protein bands were visualized with Coomassie blue staining.

### Animal studies

Female C57BL/6 mice were purchased from Jackson Laboratories (Bar Harbour, ME, USA) and maintained in an animal biosafety level 2 (ABSL2) facility. Animals were housed in microisolator cages under pathogen-free conditions with food and water available *ad libitum* and maintained on a 12 h light cycle. All animal protocols were reviewed and approved by the Institutional Animal Care and Use Committee (IACUC) of The University of Texas Medical Branch (1007037D). The mice were housed within the animal facility for at least 1 week prior to experimentation to allow adequate acclimation.

### AuNP immunization

Female 5-to-7-week-old C57BL/6 mice were immunized intranasally three times in 2-week intervals. The animals received either AuNP-EscC, AuNP-LomW, AuNP-Eae, or unconjugated AuNPs. Each vaccine formulation consisted of 5 µg of protein along with 10 µg of detoxified cholera toxin B subunit (Sigma Aldrich). Control groups received unconjugated AuNPs along with the same concentration of adjuvant. To evaluate sera antibody titers, whole blood was collected retro-orbitally 1 week prior to the first vaccination (baseline titers) and 2 weeks following the last boost (immune titers) into microvette tubes without anticoagulant. To isolate sera, blood was incubated for 30 min at room temperature (RT) to allow clotting and then centrifuged (5,000 × *g* for 5 min). Sera were removed and stored at −80°C until use. For fecal IgA titers, fecal samples were collected 1 week prior to the first vaccination (baseline titers) and 2 weeks following the last boost (immune titers). Feces were resuspended in PBS to a final concentration of 100 mg/mL, vortex homogenized, and pelleted by centrifugation to remove fecal debris. Supernatants were collected and stored at −80°C until use.

### Infection, bacterial shedding, and colonization

For initial infection studies, 10- to 12-week-old female C57BL/6 mice were infected with either DBS770 or DBS771 or mock-infected with sterile 1× PBS, following the acclimation period. For vaccine studies, mice were infected with either DBS770 or DBS771 3 weeks following the last boost. The feeding method of infection was carried out as previously described (25). Briefly, bacterial inoculums of either DBS770 or DBS771 were prepared as described earlier. A 6-µL inoculum containing approximately 1 × 10^9^ CFU of bacteria was inoculated onto a ~35 mg piece of irradiated rodent chow. One piece of the inoculated rodent chow was presented to each mouse following a 12-h fasting period. Mice were observed until full consumption of the chow to ensure infection. A piece of rodent chow was inoculated in tandem and used for dilution to enumerate infectious dose. Mice were weighed the day before infection, and every day following infection, and were humanely euthanized with CO_2_ after losing 20% of their starting body weight. Feces were collected prior to infection (day 0) and every 2 days following infection to measure bacterial shedding when possible. The fecal samples were resuspended in 1 mL PBS, serially diluted, plated onto MacConkey agar supplemented with 10 µg/mL chloramphenicol, and incubated overnight at 37°C. To assess bacterial colonization of the gastrointestinal tract, ceca and large intestines were removed from remaining mice at 14 dpi. Organs were homogenized in 1 mL sterile PBS, serially diluted, and plated onto MacConkey agar supplemented with 10 µg/mL chloramphenicol to quantify bacterial colonization. The bacterial limit of detection (LOD) was based on the lowest plated dilution and an average of the fecal or organ weights that were collected.

### Tissue collection and histology

At necropsy on day 14 post-infection, ceca and large intestines of either mock- or *C. rodentium*-infected animals were collected, placed in cassettes, and fixed in 10% buffered formalin for 24 h. They were then dehydrated and embedded in paraffin, and 5-μm tissue sections were cut and stained with hematoxylin and eosin (performed by The University of Texas Medical Branch Department of Pathology). Slides were visualized for pathology using an Olympus BX51 upright brightfield microscope.

### Fecal LCN-2 quantification

For the initial infection study, feces were collected at days 3 and 7 post-infection. For the vaccine studies, feces were collected before infection and at days 2, 4, and 6 post-infections. Fecal samples were resuspended in PBS with 0.1% Tween-20 to a final concentration of 100 mg/mL, homogenized for 10 min with vortexing, and centrifuged at 14,000 × *g* for 10 min. Supernatants were collected and frozen at −80°C until use. LCN-2 concentrations were determined using a DuoSet Mouse Lipocalin-2/NGAL ELISA Kit (R&D Systems), according to the manufacturer’s instructions.

### Detection of antigen- and pathogen-specific antibodies

Serum and fecal samples were collected from mice, and the protein- and pathogen-specific sera total IgG and fecal total IgA titers were determined by indirect enzyme-linked immunosorbent assay (ELISA). Briefly, for the antigen-specific titers, high-binding microplates (Costar Ref 9018) were coated with either recombinant EscC, LomW, or Eae (1 µg/well) in 1× sterile PBS. For pathogen-specific titers, lysates of *C. rodentium* DBS770 or EHEC 86–24 were prepared by diluting overnight cultures of bacteria 1:20 in DMEM without FBS or antibiotics and incubating statically for 4 h at 37°C to induce expression of the T3SS proteins. Cultures were pelleted and resuspended in 1 mL 1× PBS. Bacteria were inactivated and lysed by incubation at 65°C for 1.5 h. Protein concentration of lysates was determined via BCA assay, and lysates were diluted in 1× PBS and used to coat microtiter plates (1 µg/well). Coated plates were incubated overnight at 4°C. Wells were washed twice with washing buffer (1× PBS with 0.05% Tween-20) and then blocked with blocking buffer (1× PBS with 0.10% Tween-20 and 1% BSA) at RT for 2 h. The wells were washed twice with washing buffer before the addition of sample diluent (1× PBS with 0.05% Tween-20 and 0.5% BSA). The sera or feces were added to each top dilution well in duplicate, and twofold dilutions were performed, followed by a 2 h incubation. The wells were then washed three times and diluted goat anti-mouse IgG or IgA (Southern Biotech Ref 1030–05) (1:5,000) in blocking buffer was added into each well and incubated for 3 h. Plates were washed four times with washing buffer prior to the addition of tetramethylbenzidine (TMB) substrate solution (Invitrogen). Stop solution (2N H_2_SO_4_) was added to each well, and the samples were immediately read at 450 and 570 nm using a microplate reader (BioTek). The results were reported as previously described with the reciprocal of the highest titer, giving an OD reading of at least the mean +2SD compared with the baseline sera or feces (15, 16). Titers were measured for individual mice and were performed in duplicate, and results were reported as mean reciprocal endpoint titers.

### Serum bactericidal assay

Serum bactericidal assays were done as previously described, with some modifications (15, 52). Sera from AuNP-EscC, AuNP-LomW, or AuNP-Eae immunized mice (*n* = 12) were pooled and either stored at −80°C or subjected to heat inactivation (56°C for 30 min). Serum from naïve C57BL/6 mice was added to inactivated serum (1:1) as an active source of complement. *C. rodentium* DBS770 was grown and prepared as previously described to induce the T3SS proteins. After incubation in DMEM for 4 h, bacteria were pelleted and resuspended in 1X PBS, and 50 µL reaction mixtures containing 1 × 10^5^ CFU DBS770 were prepared with 10% active, inactive, or inactive serum with exogenous complement. Bacteria were incubated in the presence of serum for 1 h at 37°C with slight agitation. The negative control contained bacteria with serum from mice immunized with the adjuvant only. Following incubation, reaction mixtures were bought up to a total volume of 1 mL with 1× PBS, and viable CFU counts were determined by diluting and plating on LB agar plates with 12.5 µg/ml chloramphenicol. The serum bactericidal percentage was determined using the following equation: (bacterial CFU in adjuvant-only sera – bacterial CFU in treatment group sera)/bacterial CFU in adjuvant-only sera ⨉100. The results were obtained from two independent experiments using pooled sera from *n* = 12 mice.

### Serum adherence inhibition assay

Caco-2 cells (ATCC HTB-37) were maintained at 37°C with 5% CO_2_ in complete DMEM (Gibco), supplemented with 1 mM sodium pyruvate, 1× non-essential amino acids, penicillin, and streptomycin (100 U/mL and 100 µg/mL, respectively), and 10% FBS. For adhesion assays, 24-well plates were seeded with 1 × 10^5^ cells/well in DMEM without antibiotics and incubated overnight to form monolayers. *C. rodentium* DBS770 cultures were grown as described to induce T3SS proteins. Following incubation in DMEM, cultures were pelleted and resuspended in 1× PBS. Bacterial inoculums were adjusted to a multiplicity of infection (MOI) of 10 [1 × 10^6^ CFU (input)] and incubated in the presence of heat-inactivated sera (10%) from AuNP-EscC-, AuNP-LomW-, or AuNP-Eae-immunized mice, adjuvant-only-treated mice, or naïve mice, for 1 h at 37°C with slight agitation. After incubation with sera, bacteria were collected in 1 mL of fresh DMEM without antibiotics and used to infect the Caco-2 monolayers for 2 h at 37°C with 5% CO_2_. The cells were then washed three times with 1× PBS prior to addition of 100 µL of 0.1% Triton X-100 in PBS. After detachment, cells were serially diluted in PBS and plated on LB agar plates supplemented with 12.5 µg/mL chloramphenicol for CFU enumeration of adhered bacteria (output). The percentage of adhered bacteria for each condition was calculated as the output/input × 100 and then normalized to the percentage of adherence relative to the bacteria incubated in the presence of naïve sera. The results were obtained from two independent experiments using pooled sera from *n* = 12 mice.

### Bacteria serum recognition and fluorescence microscopy

To determine the adherence of antibodies to the surface of either *C. rodentium* DBS770 or EHEC 86–24, bacteria were grown as described to induce the T3SS proteins. Following incubation for 4 h in DMEM, bacteria were pelleted, resuspended in 1× PBS, and incubated in the presence of heat-inactivated sera (10%) from AuNP-EscC-, AuNP-LomW-, and AuNP-Eae-immunized mice and adjuvant only treated mice. After incubation, cells were washed, fixed, and stained with a goat anti-mouse IgG conjugated to Alexa-488 secondary antibody (Invitrogen Ref A32766) (1:10,000). Bacterial DNA was visualized using DAPI (Molecular Probes, Invitrogen) and mounted using ProLong Gold Antifade (Molecular Probes, Invitrogen). Slides were visualized by Zeiss LSM 880 (UTMB Optical Microscopy Core), and images were analyzed using ImageJ Software.

### Statistical analysis

All statistical analyses were done using GraphPad Prism Software (V 9.5.1). *P*-values of ≤ 0.05 were considered statistically significant. Quantitative data are expressed as means ± SD or SEM. All data were analyzed for normality before the corresponding test was run. Results of the antigen-specific antibody ELISAs were analyzed with the Mann-Whitney non-parametric test while significant differences in pathogen-specific antibody titers were determined via the Kruskal-Wallis’s test followed by Dunn’s multiple comparisons test. Significant differences in bacterial shedding, weight changes, and serum bactericidal killing were determined either via two-way analysis of variance (ANOVA) or mixed-effects analysis followed by Tukey’s multiple comparisons test. Significant differences in serum adherence inhibition or bacterial burden of feces or organs were determined via one-way ANOVA followed by Dunnett’s multiple comparison’s test.

## SUPPLEMENTAL MATERIAL

Supplemental figures spectrum.02261-23-s0001.pdf Figures S1 and S2.

ASM does not own the copyrights to Supplemental Material that may be linked to, or accessed through, an article. The authors have granted ASM a non-exclusive, world-wide license to publish the Supplemental Material files. Please contact the corresponding author directly for reuse.
