# Supplementary material for: Efficacy of EHEC gold nanoparticle vaccines evaluated with the Shiga toxin-producing Citrobacter rodentium mouse model
Source: Microbiol Spectr. 2023 Dec 4;12(1):e02261-23. doi: 10.1128/spectrum.02261-23 (PMC10783022; doi:10.1128/spectrum.02261-23)
Supplement: Supplemental figures — Figures S1 and S2. [file spectrum.02261-23-s0001.pdf]

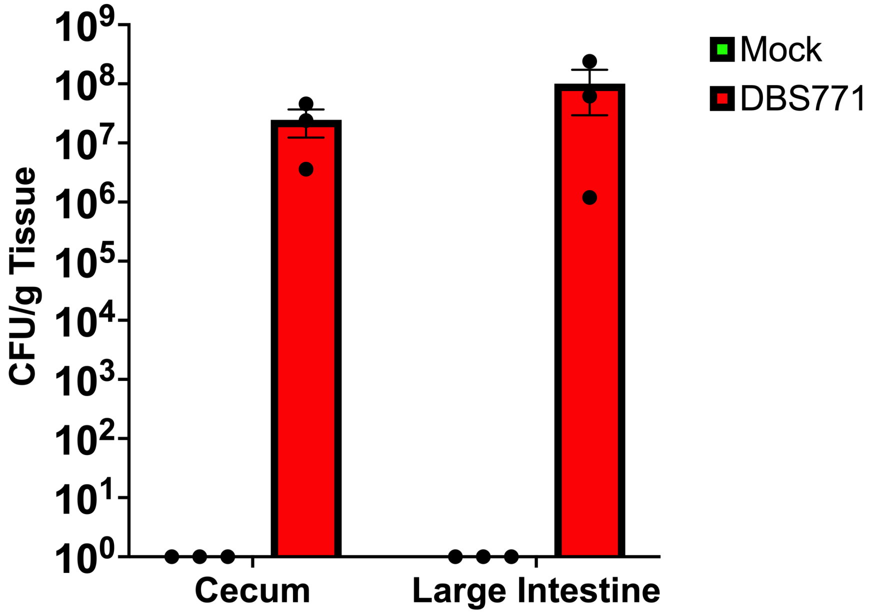


**Figure S1. Organ burden following infection of 10-12-week-old mice with Stx2d-negative *C. rodentium* via the feeding route.** Ceca and large intestines were collected from half of the mice infected with *C. rodentium* DBS771 or PBS (mock) at 14 dpi. Viable counts were expressed as CFU/g of tissue, and the average CFU (±SEM) of 3 mice is shown.

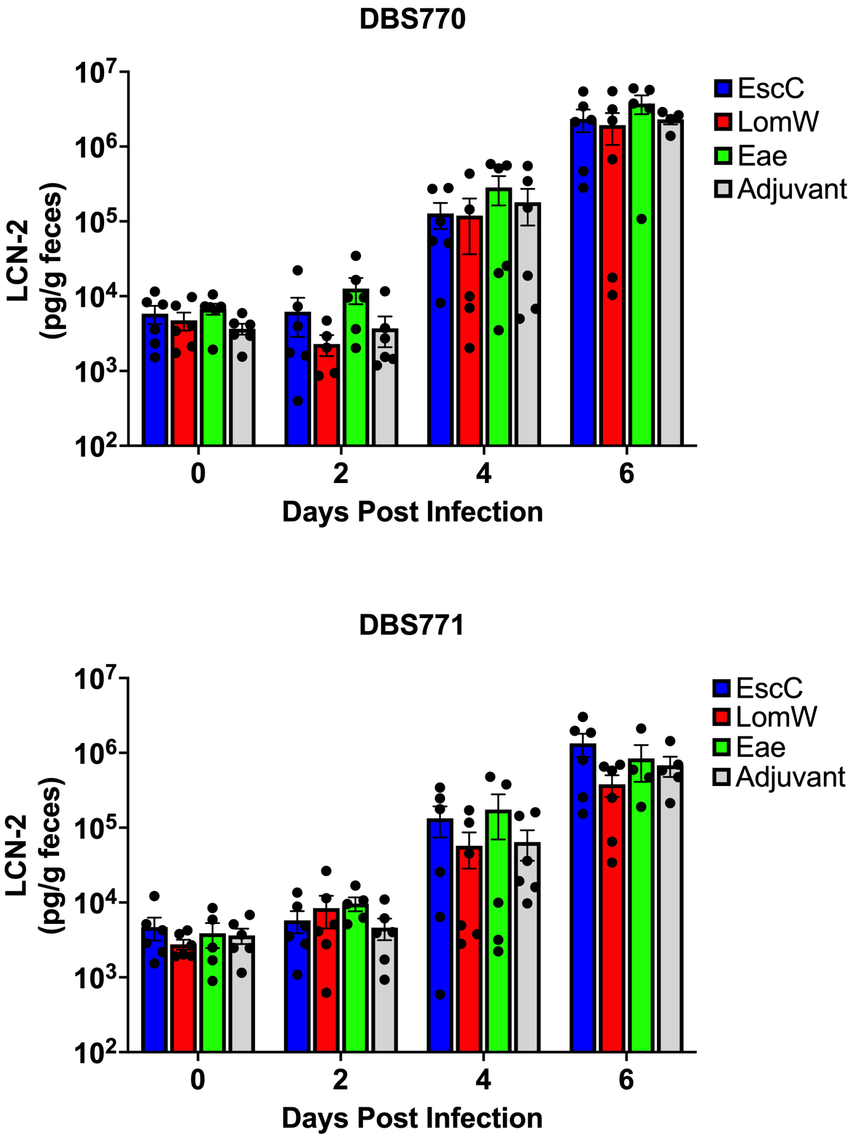


**Figure S2. LCN-2 concentrations in feces of AuNP-vaccinated mice after *C. rodentium* infection.** LCN-2 concentrations in the feces of mice that were vaccinated with either AuNP-EscC, AuNP-LomW, AuNP-Eae, or adjuvant-only treated, and then infected with either DBS770 or DBS771. Feces were collected prior to infection and at 2, 4 and 6 dpi. Each dot represents an individual mouse (starting with *n*=6 mice/group) , and the bars indicate the average (±SEM).
